# Sustainable Biomass Activated Carbons as Electrodes for Battery and Supercapacitors—A Mini-Review

**DOI:** 10.3390/nano10071398

**Published:** 2020-07-18

**Authors:** Glaydson Simões dos Reis, Sylvia H. Larsson, Helinando Pequeno de Oliveira, Mikael Thyrel, Eder Claudio Lima

**Affiliations:** 1 Department of Forest Biomaterials and Technology, Swedish University of Agricultural Sciences, Biomass Technology Centre, SE-901 83 Umeå, Sweden; sylvia.larsson@slu.se (S.H.L.); mikael.thyrel@slu.se (M.T.); 2 Institute of Materials Science, Federal University of Sao Francisco Valley, Juazeiro, BA 48920-310, Brazil; helinando.oliveira@univasf.edu.br; 3 Institute of Chemistry, Federal University of Rio Grande do Sul (UFRGS), Av. Bento Gonçalves 9500, Porto Alegre 91501-970, Brazil; profederlima@gmail.com

**Keywords:** biomass carbon electrodes, battery and supercapacitors, structure–performance relationship

## Abstract

Some recent developments in the preparation of biomass carbon electrodes (CEs) using various biomass residues for application in energy storage devices, such as batteries and supercapacitors, are presented in this work. The application of biomass residues as the primary precursor for the production of CEs has been increasing over the last years due to it being a renewable source with comparably low processing cost, providing prerequisites for a process that is economically and technically sustainable. Electrochemical energy storage technology is key to the sustainable development of autonomous and wearable electronic devices. This article highlights the application of various types of biomass in the production of CEs by using different types of pyrolysis and experimental conditions and denotes some possible effects on their final characteristics. An overview is provided on the use of different biomass types for the synthesis of CEs with efficient electrochemical properties for batteries and supercapacitors. This review showed that, from different biomass residues, it is possible to obtain CEs with different electrochemical properties and that they can be successfully applied in high-performance batteries and supercapacitors. As the research and development of producing CEs still faces a gap by linking the type and composition of biomass residues with the carbon electrodes’ electrochemical performances in supercapacitor and battery applications, this work tries to diminish this gap. Physical and chemical characteristics of the CEs, such as porosity, chemical composition, and surface functionalities, are reflected in the electrochemical performances. It is expected that this review not only provides the reader with a good overview of using various biomass residues in the energy storage applications, but also highlights some goals and challenges remaining in the future research and development of this topic.

## 1. Introduction

The conversion of biomass residues into bio-based materials can provide opportunities for biomass-based industries by reducing costs and even creating value from their by-products [1,2,3,4]. Biomass-derived activated carbons (ACs) can be obtained with tailored properties to meet the tremendous need for low-cost, high-performance, porous carbons for sustainable technologies, such adsorbents water and air purifications [5]. However, AC is one of the most common materials for fabricating electrodes in electrochemical storage devices [6,7,8].

ACs are usually in the form of powder. However, they can also be synthesised in different morphologies, such as thin films, monoliths, or fibres. Powders are commonly used for conventional electrode fabrication [6]. ACs might have a sizeable geometric surface area, which results in a low-pressure drop at high flow rates, making them very useful as adsorbents and supports for catalysts or water remediation in environmental applications [5,6,7,8,9]. ACs have also shown promising results as electrodes for batteries and supercapacitors [7,8,9]. ACs have a hierarchic pore network with large mesopores that facilitate ion transport and meso- and micropores with available sites for ion storage that enable high-performance energy storage [7,8,9].

Supercapacitors and batteries are critical enabling technologies and at the centre of vast global research, initiatives to meet the rising global demand for clean, sustainable energy [10,11,12,13]. Supercapacitors (SCs) are systems that store and deliver energy via ion electrosorption or rapid redox-processes to enable high discharge rates while batteries rely on more sluggish (bulk) redox-processes, such as ion intercalation or conversion reactions [10,13]. Compared to supercapacitors, most batteries have a higher specific energy and lower specific power, which makes them extremely suitable for mobile energy storage applications [10,11,12,13]. Supercapacitors are more employed for short-term energy storage systems or burst-mode power delivery, such as balancing grid-scale power spikes, the recuperation of braking energy, and the starting of cars, because of their elevated power density, fast charge/discharge rates, and long-lasting cycle life [10,11,12,13].

Supercapacitors and batteries are both composed of electrodes with a performance critically dependent on intrinsic properties of constitutive materials such as high surface area and electrical conductivity [7,10,11,12,13,14,15]. To date, various electrode materials, including transition metal oxides and hydroxides and conductive polymers, have been explored for high-performance energy storage devices [10,11,12,13,14,15]. In most of these systems, the carbon in some form is needed to ensure electrical conductivity considering the high resistivity of many metal oxide materials since easy electron transportation is required in the direction of the electrode-electrolyte interface. Several carbon sources such as carbon black, activated carbon, graphene, carbon nanotubes, carbide-derived carbon, or carbon aerogels have been employed as sole electrode materials or components for hybrid electrodes [9,10,16,17]. Many of these carbon materials suffer from energy- and time-consuming synthesis procedures and rely on non-sustainable carbon sources, such as by-products from the petrol industry. By employing carbon materials from biomass, it is possible to simplify the process significantly and to switch to a renewable and eco-friendly feedstock [5,9,18,19]. The facility of the employment of the biomasses for the preparation of carbon-based electrodes consists of the fact that a large variety of biomass residues can be easily found and this makes the process cheaper and easier to be implemented since costs related to the purchase/transportation of the precursors are avoided. Besides, the biomass waste-generating companies can even pay for the activated carbon/electrode companies to receive the biomass residues because this could be even lesser expensive than other treatments or landfilling process.

The utilisation of biomass to produce value-added products would have at least two simultaneous impacts, namely (i) environmental pollution control and (ii) wealth creation (through the synthesis of hi-tech materials) in an essential step in the direction to the circular economy applied in energy storage devices that preconise a “trash-to-treasure” cycle. 

For the fabrication of the energy storage devices preparation of carbon electrodes, ACs can serve, thereby, either as the sole electrode material (for ion electrosorption via supercapacitors– as a promising electron double-layer capacitive (EDLC) material) or as a tunable substrate to attach heteroatoms (O, N, H, etc.) capable of boosting their electrochemical performances [9,17,18,19,20,21]. The preparation of ACs can be done by pyrolysis and hydrothermal processes, and their quality and properties are affected by many factors, including the type of precursors, type of pyrolysis, pyrolysis conditions, and type of activation (chemical and or physical) [18,19,20,21]. Therefore, more fundamental studies are needed to optimise the properties of the final product (ACs) specifically for their applications as energy storage devices.

The current state-of-the-art presents a large gap between our ability to produce different carbon electrodes (CEs) from biomasses and how resulting properties are connected to the electrochemical performances of the CEs in supercapacitor and battery applications. This work tries to diminish this gap by explicitly focusing on the correlation of carbon properties after pyrolysis and ACs electrochemical properties resulting therefrom. Also, this work discusses that different pyrolysis methods and biomass can provide AC electrodes with optimised energy storage metrics. 

## 2. Energy Storage Devices (Battery and Supercapacitors)

The most common electrochemical energy storage devices include Li-ion batteries (LIBs) and SCs [10,11,12,13,14], which both provide huge potential and promising solutions since they can efficiently store energy from sustainable sources. The electrochemical processes occurring in SCs and batteries differ through their charge-storage metrics. LIBs, which are the most used ones, build on the insertion of Li^+^ that favours redox reactions at electrodes/electrolytes in a diffusion-controlled slow process.

A battery consists of one or more electrochemical cells [9,12,15] that are applied to store chemical energy for conversion into electrical energy. The energy is released through redox reactions that occur between both carbon or any electrode materials and the electrolytes [9,12,15]. Both devices are typically composed of electrodes, i.e., a cathode (a positive electrode) and an anode (negative electrode), as well as an electrolyte that allows ions transport, a separator that separates the two electrodes, and current collectors that allow current to flow out of the cell to perform work.

The flow of electrons is favoured by an electric current that takes place from oxidisation in the anodes, which in turn provoke a reduction at the cathode. Batteries can keep our devices working for many hours, days and weeks, due to high energy density, but, on the other hand, they can take hours to recharge when they run down [9,10,11,12,13,14,15,22] since the characteristic power density for these systems is typically low. 

For applications that need rapid power delivery and recharging, supercapacitors (also called electrochemical capacitors) are the most appropriate devices. Applications in short-term energy storage and regenerative braking, such as the recuperation of braking energy and the starting of cars, are mostly employed while the battery is mostly employed for mobile energy storage applications such as a battery for cars, computers, etc. [9,10,11,12,13,14,15,22].

Two primary mechanisms prevail in the overall energy storage in supercapacitors: the electrical double layer capacitance (EDLC) and the pseudo-capacitance. EDLC results from adsorption-dislodging of ions at an interface electrolyte-electrode. The most promising materials applied as EDLC candidates are carbon allotropes. Devices based on EDLC materials are characterised by high power but low specific capacitance. On the other hand, the pseudo-capacitance is favoured by redox reactions at the surface of electrodes classified as a faradaic process. Despite the low conductivity of some pseudocapacitive (metal oxide layers), conducting polymers (CPs) present outstanding electrical conductivity properties that enable their use as a binder for SCs. As for disadvantages, the low stability and poor mechanical properties of CPs can be considered. To circumvent these drawbacks, the development of hybrid materials based on carbon derivatives and pseudocapacitors tends to synergically reinforce the potential of both components (EDLC and pseudo-capacitance), as previously reported in [23,24,25]. 

EDLC prototypes store charges by adsorbing electrolyte ions onto the electrode’s surfaces [10,12]. There are no redox reactions to make them work, so the response to changes in potential without diffusion limitations is rapid and leads to high power [10,12]. However, the charge is contained on the surface, so the energy density of EDLCs is lesser than that of batteries [10,12]. 

Batteries and SCs rely on electrochemical processes, although each has and work through different mechanisms that determine their relative energy and power density. Traditionally, batteries and SCs work as symbiotic devices. The SC readily transferred energy, while the storage capacity of a rechargeable battery filled the needs of a power bank [9,10,11,12,13,14,15,22]. Both devices seemed to be unparalleled in their respective fields. The supercapacitor exhibits huge power density, while the battery presents very high energy density. Recently advanced supercapacitors have come to market that breaks down that barrier. It is worth mentioning that different strategies have been considered to improve the energy in supercapacitors. In addition to the above-described development of hybrid composites (EDLC + pseudocapacitance), which are responsible for an improvement in the specific capacitance (Spe.Cap) of the device, the electrochemical window is another critical parameter for improvement in the energy density ($E=\frac{1}{2}CV^{2}$). The higher potential window has been successfully reached from an asymmetric arrangement of electrodes (battery-like and SC-like electrodes), allowing that potential window can be higher than 1 V.

Porous carbons are widely desired and employed as efficient electrodes due to their large specific surface area (SSA), well-developed porosity, and pore-size distribution, which can be tailored for a more suitable structure to the size of the electrolyte ions providing higher conductivity, and good physicochemical stability. Furthermore, the AC’s porous surface can be modified with some functionalities that can improve their electrochemical performances, which is explained and discussed later in Section 5.3. 

## 3. Biomass Carbon Sources and Composition as Raw Material for Carbon Electrodes (CEs)

Carbon is one of the most critical elements for humankind. It is essential in people’s lives as well as for industrial processes as a raw material [2,5,22]. Due to its diverse electronic properties, carbon materials have a wide range of structures and properties according to their C–C bonding [2,5,22]. In light of these statements, strategies for the development of carbon materials such as nanofibers, graphene, graphite, etc., have been implemented which have successfully resulted in developments in carbon science applications and technology [5,7,8,26]. It can be said that carbon materials almost include the properties of all the materials on the earth, such as the hardest and softest, insulators, adsorbents, conductors and semiconductors, thermal conductors, and insulators, etc. [2,5,22]. With the evolution of science and technology, carbon utilisation seems to contain unlimited possibilities of turning it into useful materials and development.

Another essential aspect is that sources of carbons can be easily found everywhere in the world, mainly from forest and animal residues, as so-called biomass. International Union of Pure and Applied Chemistry (IUPAC) defines biomass as material produced by biological growth (plants, microorganisms, animals, etc.) [27], It is also an applied term to the use of these biomaterials for energy production (heat or electricity), or in various industrial processes as a raw substance for a range of products [27]. Unlike fossil fuel, biomass can be considered a renewable material because its inherent energy depends uniquely on the sun to grow and can regrow in a relatively short time.

Each year, billions of metric tons of organic residues are generated all over the world from activities of farming and crop production, food industries, animal husbandry, etc., requiring tremendous efforts to develop systems in which production, conversion, and utilisation of these residues are carried out efficiently and under environmentally sustainable conditions [28,29,30,31]. Therefore, it is imperative to explore and employ renewable and natural sources of energies to replace fossil sources, encouraging us to seek greener and more efficient energy technologies to meet the increasing demands for energy and eco-friendly materials [28,29,30,31].

It has been shown in the literature that there are a variety of materials synthesised from heterogeneous biomass precursors [1,3,4,5,6,7,8,9,17,18,19,20,21,32]. These carbon materials might exhibit different properties and structures that are in function of the biomass type and initial composition. Many reports in the literature correlate the properties of the selected biomasses with the electrochemical metrics of CEs made from them.

The composition of the biomass can play an essential role in the performance of CE for energy storage devices since it will influence the properties of the final AC properties. Table 1 shows the main components of several biomasses in terms of hemicellulose, cellulose, and lignin. Biomass rich in cellulose can be successfully employed to synthesise cellulose-based carbons for electrodes [35,36]. During the thermal treatment of the biomass precursors, hemicellulose, cellulose, and lignin decompose at different rates and within distinct temperature ranges [4]. While lignin is pyrolysed over an extensive temperature range and shows the behaviour characteristic of solid fuels, hemicellulose and cellulose decomposition is sharp in a narrow temperature range [4]. These differences certainly provide ACs with different properties, which also lead to CEs with different electrochemical metrics.

Nevertheless, the carbon content in cellulose-rich materials can reach 50% [34]. However, after carbonisation, this content may increase above 80% or even 95% for ACs, which is interesting since it can lead to an elevated and developed porosity [37]. The high cellulose content also plays a crucial role in developing mesopore structure in ACs while lignin, for example, can promote the formation of layered structure and maximisation of micropores during the preparation of AC [38].

Zhuo et al. [39] fabricated CE from activated carbon made from cellulose, (1364 m^2^ g^−1^) with excellent electrochemical performance. It presented a Spe.Cap of 328 F g^−1^ at 0.5A g^−1^ as well as outstanding cycling stability with 96% of the capacitance retention after 5000 charges/discharge cycles. In another work [40], micro/mesoporous carbon was successfully obtained from cellulose and used to make CE for supercapacitors. The fabricated CE displayed specific capacitances of 160 F g^−1^ at 0.2 A g^−1^ and also exhibited very excellent cycle stability.

Lignin-rich materials are also useful as alternative raw precursor materials for CE preparation [41]. In typical biomass, lignin links between cellulose and hemicellulose. Tian et al. [41] fabricated electrodes by using lignin as primary raw material and reported that due to its high SSA and wide pore size distribution, the derived lignin electrode exhibited high specific capacitance equal to 328 F g^−1^ at 0.2 A g^−1^. It also presented very good cycling stability (97% capacitance retention after 10 000 cycles). Also, the derived lignin electrode delivered a high energy density (6.9 W h kg^-1^) at 50 W kg^−^^1^. Lin et al. produced lignin-based porous carbon by simple chemical activation with KOH followed by pyrolysis [42]. The obtained CE presented good capacitance performance (165 F g^-^^1^ at 0.05 A g^-^^1^) and outstanding cycling stability (97% over 5000s). The CE also exhibited an energy density of 5.7 Wh kg^−1^ at a power density of 15 W kg^−1^.

Hemicellulose can also be an attractive raw material for AC preparation for making CE. For instance, Wang et al. [43] extracted hemicellulose from hemp stem and treated it hydrothermally followed chemical activation with KOH. The obtained hemicellulose-derived CE exhibited excellent electrochemical performance (capacitance of 318 Fg^−1^), which is attributed to abundant micropores and oxygen functionalities. 

## 4. Thermal Process for Carbon Electrodes Preparation and Heating Process Considerations

It is well reported that several pyrolysis methods can be employed to prepare useful ACs [43,44]. However, conventional pyrolysis, hydrothermal carbonisation (HTC), and microwave heating are the most used ones, and all of them can yield AC with exciting properties. These methods present different ways of heating inside the reactor; conventional pyrolysis and hydrothermal occur via conduction and/or convection while microwave occurs via electromagnetic waves [45,46]. The heating in microwaves generates fewer energy losses to the environment and consequently has more considerable energy savings when compared to conventional pyrolysis [45,46].

**Figure 1:**
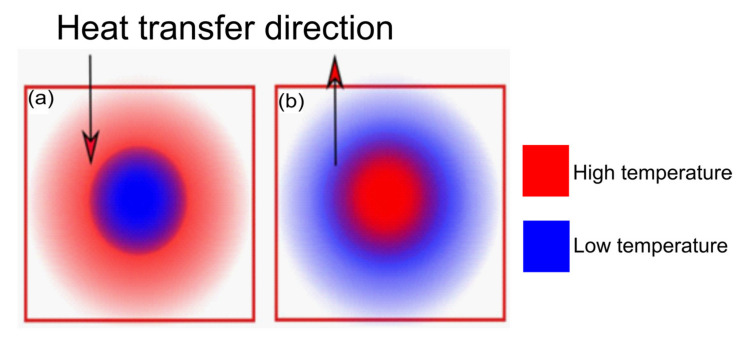
- Schematic diagram of temperature distribution, in the (**a**) conventional pyrolysis and HTC and (**b**) microwave heating.

Compared to the conventional process, microwave heating is directly related to the internal heating of the material. In this sense, energy is transferred from the interaction between molecules or atoms, representing the transformation of electromagnetic energy in thermal energy. In contrast, in heating processes based on conduction, convection, and radiation, heat is transferred from the surface of the material towards the centre. Figure 1 schematises how the heating profiles move concerning the material.

Since microwaves are capable of penetrating the material that will then retain this energy, heat is generated throughout the sample [46,47]. The uniformity of the heating will depend on the sample size and microwave penetration depth [46,47].

Some advantages of microwave heating when compared to the conventional heating are related to the fact that microwaves provide the heating of the material in the absence of contact, the energy (heating) is transferred with speed and uniformity and at the same rate of volumetric heating, and has a high level of safety and automation potential [46,47]. Additionally, such a system offers advantages of fast start-up and processing execution and high energy efficiency (in terms of the amount of energy involved in the quantity effectively absorbed) [46,47].

HTC process is usually carried out in subcritical water at much lower temperatures when compared to other pyrolysis methods. The biomass or any precursor is heated in a hermetic reactor under the autogenous pressure [48]. However, when other methods are compared with HTC, conventional pyrolysis presents some advantages such relatively simple process which is more mature and easier to be industrialised while the microwave is still being tried to be adapted to be industrialised. However, HTC has several advantages. For instance, since the carbonisation reaction is carried out in the water, no drying process is required for HTC. Moreover, using HTC the ash content, in the AC composition, increases (as is with all the carbonisation methods) but to a lesser extent due to the continuously washing condition into the liquid phase, and this can improve the AC electrochemical performances since ash can hinder it [49].

However, in terms of which method is the most appropriate for producing ACs with improved and better characteristics, there is not yet a categorical conclusion to be drawn. In the international literature, there are several controversial takeaways and conclusions. The right thing to do should be to analyse each case based on the type of biomass and what the application of the produced ACs because what influences the final quality of an AC is a set of factors and not just the method of heating itself, such as operating conditions and type of raw material that is being used [46,47].

For instance, Hoffmann et al. [50] prepared ACs (by HTC) from potato residues and applied them as carbon electrodes (CE) for SCs. They found that the HTC method provided AC with very high carbon content, and this reflected in good electrochemical performance (Spe.Cap of up to 134.15 F g^−1^). They reported that pseudocapacitive effects explain the relatively high capacity due to the high O-content in the carbon (8.9 wt.%).

In another work, Liu et al. [51] produced ACs from palm residues by microwave method, which yield ACs with very high specific surface area (SSA) (344 m^2^ g^−1^). The microwave carbon displayed high Spe.Cap of 226.0 F g^−1^ at 0.5 A g^−1^ as well as an excellent performance on a charge-discharge process with an energy density of 72.3 Wh kg^−1^ at a power density of 1.4 kW kg^−1^ and 50.0 Wh kg^−1^ at 28.8 kW kg^−1^.

Conventional pyrolysis is by far the most applied method for preparing CEs. Hou et al. [52] used rice biomass to fabricate CE, and the final materials also exhibited interesting electrochemical properties, e.g., the Spe.Cap of 218 F g^−^^1^ at 80 A g^−^^1^ in 6 M KOH and high energy-density of 104 Wh kg^−^^1^ (53 Wh L^−^^1^) by using ionic electrolytes.

These studies suggest that whatever method is used to prepared ACs and CEs, they can successfully be employed to make efficient CE with improved electrochemical properties. 

## 5. Preparation of ACs Through the Chemical Activation Process

The preparation of ACs usually consists of employing a thermal treatment followed by further activation (physical and/or chemical) that can take place either in a single or two-stage process. 

In the chemical activation step, the biomass is combined and mixed with chemical reagents (ZnCl_2_, KOH, NaOH, H_3_PO_4_, K_2_CO_3_, and FeCl_3_) at desired ratios to obtain ACs with desired properties [9,28,29,30,31,44,45,46,53,54,55,56]. This step is essential in which the pyrolysis and activation are simultaneously performed which might have a significant influence in the pyrolytic decomposition of the precursor and, therefore, resulting in the development of highly porous structures and functionalities on the carbon surface [54,55,56,57]. ZnCl_2_ and KOH are the most employed chemical reagents for preparing ACs [54,57]. The chemical activation step has the advantage of producing ACs with high developed porosity and elevated SSA.

**Figure 2:**
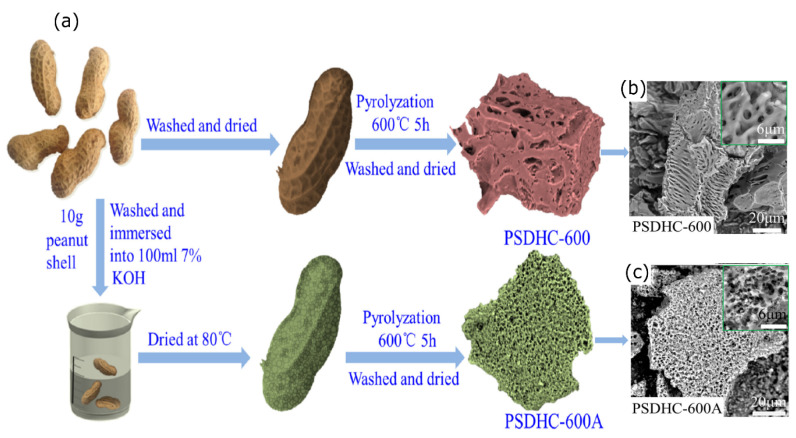
(**a**) Effect of the chemical treatment on the AC properties, (**b**,**c**) SEM images of the ACs. Figure reproduced and adapted from reference [57] with permission from Elsevier.

Lv et al. [57] reported the preparation of ACs with and without the activation of peanut shell using KOH. Thereby, it was found out that the KOH activation induces the higher number of nanoscale pores before pyrolysis (see Figure 2). 

Due to the capillarity infiltration of KOH liquid, a number of micro and mesopores can be developed into the biomass structure, thus the pyrolysis of which leads to a much finer porous structure of PSDHC-600A when compared to PSDHC-600. The finer porous structure facilitates the penetration of the electrolyte, which reduces the ion diffusion distances, providing more and efficient sites for ions storage.

Chen et al. [58] prepared ACs using tobacco stem as a precursor by mixing KOH, K_2_CO_3_, and ZnCl_2_ as chemical activation reagents. The effects of the impregnation ratio and activating agents were evaluated on AC structures. The properties of the ACs were better developed by using ZnCl_2_. Moreover, it yielded ACs with various oxygen, hydroxyl, and ester functional groups on ACs surfaces and excellent thermostability.

**Figure 3:**
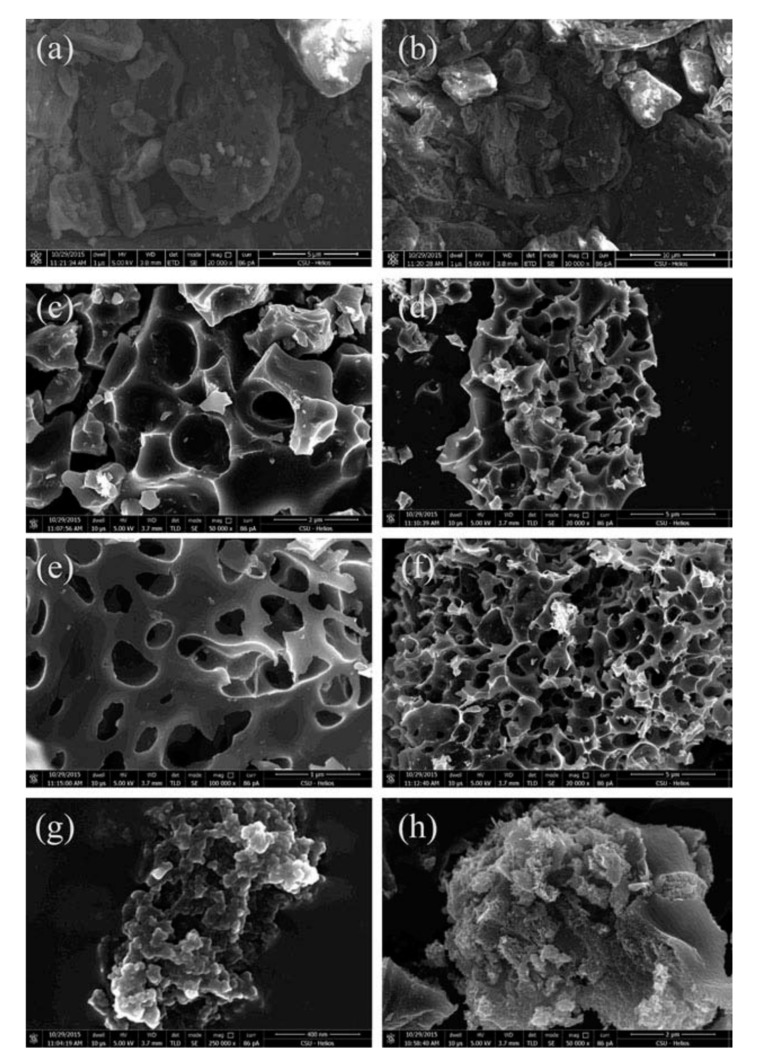
SEM images of (**a**,**b**) raw tobacco stem and AC samples: (**c**,**d**) activated with KOH, (**e**,**f**) activated with K_2_CO_3_, and (**g**,**h**) activated with ZnCl_2_ [58]. Figure reproduced from reference [58] with permission from Taylor & Francis.

The difference of the chemical reagents in carbon structures is further observed in Figure 3. It shows significant differences in their morphologies (between raw materials and AC samples). The tobacco biomass displayed a rough surface with tiny porosity (see Figure 3a,b), while AC surfaces presented massive amounts of pores structures that were created by the chemical activation (see Figure 3c–g). 

There are also observed significant differences between the ACs prepared by the different activation agents. Different pore sizes and shapes could be observed mainly for those ACs prepared with KOH (see Figure 3c,d) and K_2_CO_3_ (see Figure 3e,f), presented certain similarity in their morphologies. However, comparatively, the activation with ZnCl_2_ yielded AC with sponge-like morphology (see Figure 3g,h), and much smaller pore sizes which resulted in a much higher SSA.

Dos Reis et al. [56] prepared ACs from sewage sludge by comparing chemical activation reagents (KOH and ZnCl_2_). The KOH-treated AC obtained the smaller SSA (186 m^2^ g^−1^) than the ZnCl_2_-prepared carbon (192 m^2^ g^−1^). However, the KOH AC presented a higher presence of functional groups on its surface, and this would probably influence the electrochemical performance, since, for instance, nitrogen, oxygen, and phosphorous functionalities can enhance the electrochemical effects by improving the wettability of porous carbon in contact with electrolytes [55,59,60,61,62,63,64,65].

### 5.1. Effect of the Physical Characteristics of Biomass Carbon Electrodes for Lithium-Ion Battery (LIBs)

Ion diffusion is of considerable importance to the electrochemical performances of the CEs, mainly in the charge–discharge processes of batteries [12,14,52]. In this sense, CEs from biomass with high surface area and developed porosity is highly desired for LIBs because it can diminish the Li-ions diffusion pathways and to optimise a large electrode/electrolyte interface, which is beneficial for electrochemical reactions and therefore improving its metrics [59,60,61,62,63,64].

In this sense, materials used as electrodes might play a crucial role in the whole energy storage systems [63,64,65]. Graphite and graphene are some of the most popular anode material for LIBs [61]. However, the Li-storage capacity of graphite is not high enough to meet the demand of electric devices. However, the graphite has low both capacity (372 mA h g^−1^) and the rate performance, which is difficult to commercially popularise [61].

Thus, to enhance Li-storage capacity, efforts have been made to reach the application of porous ACs from biomasses for electrodes fabrication [66,67,68,69,70,71,72]. Many kinds of research have been devoted to the feasibility of preparation of CEs from biomasses, mainly due to the significant existence of micro-, meso-, and macropores that have huge effects on their electronic structure and electrochemical performance, such as reduced diffusion length for Li^+^ ions and electrons and improved reversible capacity (RC).

Another important consideration is that porous carbons can be easily prepared through thermal, physical, and chemical activations. These activation methods can provide carbon materials with various structures and textures, morphologies, crystallinities, and electronic features which are desirable features for CE fabrication and energy storage devices.

Some main electrochemical metrics such as initial Coulombic efficiency, rate capability and RC are compared in Table 2 to evaluate the efficiency of the carbon electrodes from biomasses for LIBs. It is observed that the biomass carbon electrodes with higher SSA are inclined to exhibit higher Coulombic efficiencies. For instance, Selvamani et al. [62] found an initial coulombic efficiency of 90% with a CE with an SSA of 1980 m^2^ g^−1^ and Hernández-Rentero et al. [63] found an efficiency higher than 99% for a CE with SSA of 1662 m^2^ g^−1^ while Lotfabad et al. [64] found a coulombic efficiency of 55% for a CE with an SSA of 130 m^2^ g^−1^ (see Table 2). However, Zhang et al. [61] fabricated a CE from rice straw and, among the reports shown in Table 2, it exhibited the highest SSA but not the best electrochemical performances.

Many reports in the literature indicate the role that SSA plays in the electrochemical process, although it is not the only parameter to be considered [52,59,65]. In terms of physical features of the CE, the combination of macro-, meso-, and micropores might enhance the CE performances in terms of i) electrode wettability and electrolyte accessibility which are mainly facilitated by the presence of macro- and mesopores [61,65], although the hydrophobicity of the CE also influences the wettability process [65,66] ii) ion diffusion rate, and iii) charge transport kinetics at the CE/electrolyte interphase which is mainly potentialised by the presence of the micropores [60]. Therefore, together with the high specific surface area, the simultaneous presence of various types of pore sizes into the CE allows for the enhancement of their electrochemical performances in energy storage devices [60,65,66,67,68,69,70,71,72,73].

Zhang et al., [66] evaluated porosity effect on the electrochemical metrics of CE for LIBs and found exciting results. They reported that the excellent electrochemical performances were reached because of the high presence of mesopores. They related that the dilated interlayer spacings (0.387–0.395 nm) positively affected the Li^+^ intercalation.

**Figure 4:**
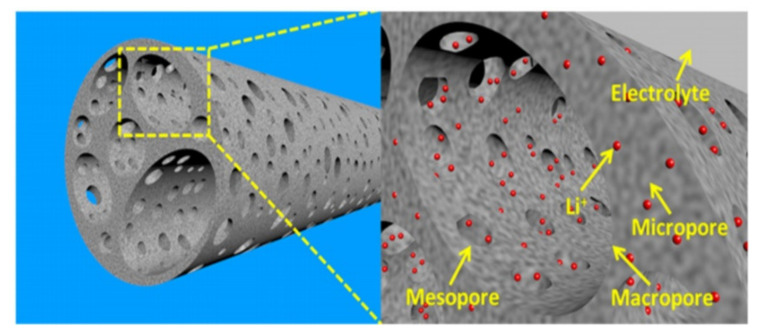
Scheme of the porous structure and Li^+^ storage system. Figure reproduced from reference [67] with permission from Royal Society of Chemistry.

Also, the high SSA maximised the electrode/electrolyte interfaces for the charge-transfer reaction. Moreover, a large amount of ordered mesoporous in the AC structure can serve as Li^+^ deposit and also potential pathways for diffusion of electrolyte which speedy the kinetic process of ions diffusion in CE surfaces and structures, leading to better electrochemical performance metrics (as illustrated in Figure 4).

**Figure 5:**
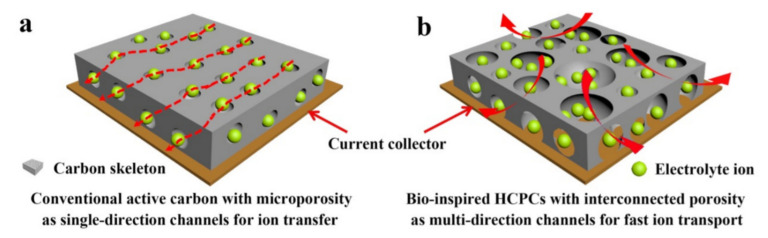
Scheme of different ion channels in (**a**) conventional AC with single direction porosity and (**b**) HCPC with multidirectional porosity for ion transfer. Figure reproduced from reference [68] with permission from Americam Chemical Society.

The effect of carbon structure was studied and highlighted by Peng et al. [68] using *Moringa oleifera* leaves as a precursor to produce highly porous AC (HCPC) with multidirectional porosity that was used as CE for efficient DLCs (see Figure 5).

Figure 5a shows conventional AC with dominant microporous structures with single-directional ion channels, which provides more reduced accessibility for electrode ions to transfer into the internal pore structure and thus resulting in poor electrochemical performances. Compared to the conventional commercial AC, the prepared highly porous N-doped AC (Figure 5b) exhibited improved characteristics and advantages, such as: (i) highly crumpled and wider pores on its surface structure promote an enhancing in the ion adsorption due to the increase of the number of active sites which maximise the useful storage of the charges; (ii) a large number of interconnected macropores might act as the ion deposit to storage electrolyte ions and enhance the access and availability of the interface between electrolyte and CE; (iii) high multidirectional porosity can also promote important channels for rapid and efficient ion transportation and transference; (iv) by increasing O- and N- atoms on AC surface enhance the wettability and the conductivity of electrons, which leads to advantage gains in the rapid charge transfer.

### 5.2. Biomass Carbon Electrodes for Double Layer Supercapacitors (DLCs)

Supercapacitors or electrochemical double-layer capacitors (DLCs) can store and deliver the electrostatic charge through ion adsorption on the surface of the electrically conductive porous CEs [72,73]. Thus, CEs with very high specific surfaces and appropriate pore distribution can also achieve very high capacities and are the critical factors for DLCs to provide active sites and channels to maximise the contact between interface CE/electrolyte and increase the adsorption of electrolyte ions [74,75].

Because of its high power density and long cycling life, DLCs have attracted very much attention over the last years, in comparison to LIBs and fuel cells [76]. However, some limitations hinder their practical applications, such as low-energy densities (~8 Wh kg^−1^) [77]. Therefore, to fulfil the essential demands of energy, novel and efficient CEs must be designed for highly efficient DLCs through simple, low-cost, and environmentally sustainable technologies routes [78].

Advanced carbon materials including graphene, carbon nanotubes, and templated carbons are the most commonly employed CE materials for commercially efficient DLCs due their useful cycle lifetime, high specific capacitance and high maximum power density [79,80,81]. However, high energy and costs are required to obtain these materials. Moreover, the process of their preparation is complicated, which makes it difficult for large-scale production [9,12].

Therefore, it is imperative to find ways and materials to fabricate lower cost and efficient CEs with comparable electrochemical performances from ample and renewable natural resources for SCs applications, and biomasses constitute one of the solutions.

Vast and exciting reports are shown in the literature concerning the production of bio-based CEs from different biomasses by applying different thermal, physical and chemical treatments to apply them in SCs application, as shown in Table 3. From these studies, different takeaways and outcomes have been achieved regarding CE for SCs which some of them were that (i) high carbon content, graphite-like and microporous structures positively influence the electric conductivity [70,81,82,83,84,85,86,87] and (ii) high electric conductivity leads to better electrochemical performance and high capacitances [64,82].

Xia et al. [88] developed porous CEs by pyrolysing O-rich biomass (sodium alginate). At the optimal experimental condition, the CE delivered a high Spe.Cap of up to 424.6 F g^−1^ in 6 M KOH electrolyte at 1 A g^−1^. The CE also presented good cyclic stability with the capacitance retention of 90% after 20,000 charge-discharge cycles. The excellent electrochemical performance of the CE was attributed to both hierarchical macro-/meso-/micro porous structures as well as the abundant presence of oxygen functional groups.

In another study, *Moringa oleifera* leaves were used as raw biomass for CEs preparation [86]. The fabricated CEs showed very high electrochemical performances such as capacitance retention higher than 90% when the current density is increased from 1.0 to 50 A g^−1^ and efficient cycling stability over 20 000 cycles. Also, CEs resented the high specific energy of 21.6 Wh kg^−1^.

He et al. [83] also used biomass residues from peanut shells to prepare CEs for SCs. The ACs were prepared by using ZnCl_2_ chemical activation followed by microwave pyrolysis. It was found that the SCs made from it displayed a high energy density of 19.3 Wh kg^−1^ at a high power density of 1007 W kg^−1^ in 1 M Et_4_NBF_4_/PC electrolyte, and a Spe.Cap of 99 F g^−1^, highlighting that the employed method of preparation was highly efficient to produce CEs with high electrochemical performance for SCs.

### 5.3. Effect of Functional Groups on Electrochemical Performances of the CEs

Functional groups on surface carbons are influenced by the precursor materials as well as the activation process [96,97]. The presence of functional groups is related to the degradation performance and ageing of AC in organic electrolytes [98,99,100]. Therefore, to diminish this degradation, it is recommended to produce functional group-free ACs [99,100]. However, the same functional groups that hinder the electrochemical performances in organic electrolytes can be beneficial in non-organic electrolytes by providing extra capacitance through a pseudocapacitive mechanism [100,101].

Furthermore, surface-functional groups including nitrogen, oxygen and phosphorous can considerably increase the electrochemical effects as well as improving the wettability of porous carbon with electrolytes, increasing so the electrochemical performances of the electrodes [102,103,104].

Ding et al. [100] related that an appropriate number of functional groups on the CE surface can enhance the electrochemical stability window, which can be reflected by high energy and power density. It is also related that the presence of certain functionalities (e.g. O–C=O or C=O) induced higher capacitance to carbon materials and that the proper functional groups hinder the potential shift of the CEs.

However, an elevated amount of functionalities on the CE surface is reflected in a high quantity of irreversible redox products that remains inside ACs’ pores, resulting in a faster capacitance fade concerning the ACs with lesser functional groups. Cao et al. [104] related that the amount of O- functional groups play a significant influence on the electrochemical SCs efficiency having CE as electrodes.

Widmeir et al. [105] prepared different carbons and concluded that the surface functionality could strongly affect the initial open-circuit voltage (OCV) and the potential shift of the CEs through the time. The large quantity of O-groups explained the high initial OCV of AC (modified by HNO_3_), while the lower OCV of AC (reduced by Ar/H_2_) was due to lesser presence of O-groups on AC structure.

Elmouwahidi et al. [106] prepared ACs using argan seed shells as a biomass source through KOH active reagent. They evaluated the effect of the presence of functional groups on electrochemical performances. They concluded that O-rich ACs exhibited the lowest Spe.Cap (259 F/ g at 125 mA/g) and capacity retention (52% at 1 A/g), because of the presence of surface carboxyl groups that hindered electrolyte diffusion into the CE pores.

On the other hand, they also concluded that N-rich ACs presented the highest Spe.Cap (355 F/g at 125 mA/g) as well as highest retention (93% at 1 A/g), because of the pseudo-capacitance effects of N functionalities.

Yang et al. [107] applied four carbons with different oxygen and nitrogen contents as well as different porosities. The effect of both functional groups and porosity were evaluated on the electrochemical properties of these carbons. It was found that the electrochemical activity increased in the order of oxygen contents.

They also found that even the carbon with the largest surface area but lower oxygen content had much lower specific capacitance than samples with higher oxygen contents, which suggests the importance of oxygen functional groups on electrochemical properties of the carbon electrodes because the oxygen groups can enhance electrolyte wettability and reactions in aqueous electrolytes.

### 5.4. Biomass Carbon Electrodes for Pseudo-Capacitance/ EDLC Hybrid Devices (HSCs)

An important procedure to reinforce the electrochemical performance of energy storage devices refers to the association of characteristic adsorption of ions by EDLC prototypes with faradaic reactions from pseudocapacitors. The incorporation of pseudocapacitive components into biomass-based carbon electrodes for the production of efficient SCs and batteries takes place from the development of asymmetric devices (strategy to improve the operating voltage window from the use of complementary working potential electrodes) or exploring the carbon-based structures as a support for the growth of pseudocapacitors layers. In the last case, the outstanding properties of biomass-based carbon electrodes (such as high surface area, porosity and electrical conductivity) can be conveniently explored to create nucleation sites for pseudocapacitors growth with an integration level required to reduce the interfacial resistance [108,109].

The production of biomass-derived carbon structures and transition metal oxide composites makes use of the porous structure of carbon derivatives to incorporate seeds and to provide the growth of metal oxide structures based on materials such as cobalt oxide, iron-oxide, nickel-cobalt, etc.

However, the typical low conductivity of resulting structures is a barrier that has been successfully circumvented by the development of composites with conducting polymers due to their high intrinsic conductivity of candidates such as polypyrrole and polyaniline. Despite these superior properties, the low mechanical resistance and low cycle life of CPs-based supercapacitors require a strong interaction of a covering layer with carbon-based systems to reach a desirable energy density in all-solid devices [110].

Arthisree and Madhuri [111] reported the development of green synthesised graphene quantum dots and polyaniline reaching high current density (670 mAg^−1^) and a specific capacitance of 105–578 Fg^−1^cm^−2^. Yu et al. reported a two-step process for the synthesis of composites based on biomass-derived 3D aerosol loaded with polypyrrole particles in supercapacitors with an areal capacitance of 419 mF cm^−2^ [112].

## 6. Future Perspectives and Current Challenges

The development of renewable carbon materials represents a “sustainable way” to the energy storage-based industry. However, the challenge in this process involves a reasonable degree of complexity that represents a multifactor correlation process involving porosity, conductivity, and flexibility for the resulting device. The production of genuinely flexible energy storage devices is a requisite for wearable electronics, and it can be considered as a process that depends on the substrate preparation procedure in which the electrical response must be nearly invariant under mechanical efforts. On the other hand, the performance of the energy storage devices depends on the available surface area and good conductivity level for charge transport. In general, the increase in the porosity yields a decrease in the electrical response (conductivity) of devices. As a consequence, the design of this 3-factor problem requires a more sophisticated solution in which the optimal condition for each factor can be far from the best performance for the final device. The design of renewable carbon material-based energy storage devices must consider a multifactor study in which the correlation of these properties is critical for the overall response of supercapacitor/battery.

## 7. Conclusions 

Enormous efforts need to be focused on developing new methods to produce materials more sustainably to ensure a smooth shift to a sustainable society. Regarding energy storage, tremendous advances have already been achieved in producing bio-based electronics. For such applications, biomass plays a significant role due to its inherent structural and chemical diversity. The literature has shown a variety of materials synthesised from heterogeneous biomass precursors for energy storage devices based on carbon electrodes. There is a lack of systematic studies correlating the electrochemical performance with the precursor physical-chemical characteristics.

This study provided insight into using suitable biomasses for the fabrication of CEs with efficient electrochemical performances for battery and supercapacitors. An in-depth comparison with the literature showed that biomass residues are already a reality in being promising candidates for high-performance carbon electrodes for battery and supercapacitors. 

For instance, physical and chemical characteristics of the CEs reflected in parameters such as the effects of porosity, chemical composition, and surface functionalities on the electrochemical performance are also affected by the intrinsic conductivity of the resulting material. The influence and role of the different biomasses and their components during the thermochemical treatment (conventional pyrolysis, microwave process, and HTC) as well as their experimental conditions. Synergies between the types of biomass and their compositions on the fabrication of carbon electrodes have essential influences on their electrochemical performances.

Biomasses can provide ACs with very high SSA and developed porosity with different pore structures which make them very suitable for electrochemical applications. The quality of the activated carbons can be determined by a proper pyrolysis method as well as the conditions that can optimise the properties of the final ACs and CEs.

## Figures and Tables

**Table 1:** Main composition of some agricultural biomass and residues.

Biomass Precursor	Hemicellulose (%)	Cellulose (%)	Lignin (%)	Ref.
Coconut husk	23.7	0.52	3.54	[33]
Sugarcane bagasse	27–32	32–44	19–24	[34]
Hardwood stem	24–40	40–50	18–25	[34]
Softwood stems	25–35	45–50	25–35	[34]
Sunflower shell	34.6	48.4	17	[34]
Bamboo	15–26	26–43	21–31	[34]
Banana residues	14.8	13.2	14.0	[34]
Cocoa pod husks	37	35.4	14.7	[34]

**Table 2:** Electrochemical performance of some representative biomass residues derived CEs for Li-ion batteries.

Biomass Precursor	Pyrolysis Method and Optimal Condition	SSA (m^2^ g^−1^)	Main Electrochemical Findings	Electrolyte	Ref.
Cattle bone	Heated at 1100 °C in for 1 h under N_2_ and washed with 1 M HCl	2096	Electrode exhibited remarkable RC of 1488 mA h g^-1^ after 250 cycles at 1 A g^−1^ and 661 mA h g^−1^ after 1500 cycles at 10 A g^−1^; at 30 A g^−1^ it delivered 281 mA h g^−1^ of RC.	1 M LiPF_6_ in DEC:EC solution (ratio 1:1)	[9]
Peanut shell	Pyrolysed at 600 °C for 5 h and immersed in a solution of containing 7%wt ZnCl_2_, 7%wt K_2_CO_3_ and 1 M H_3_PO_4_ for 48 h	706.1	Initial coulombic efficiency of 48.6% at 1 A g^−1^; RC of 1230 at 50 mA g^−1^; Rate capability of 310 mAh g^−1^ at 5 A g^−1^.	1 M LiPF_6_ dissolved in EC:EMC (1:1 *v*/*v*)	[57]
Rice straw	Heated at 400 °C for 3 h under N_2_. After, it was soaked in a KOH for 1 day. Afterwards, heated again at 750 °C for 2 h under N_2_	3315	Initial coulombic efficiency of 48% at 37.2 mA g^−^^1^; RC of 986 of 1st cycle at 37.2 mA g^−^^1^; Rate capability of 257 mAh g^−^^1^ at 0.744 A g^−^^1^.	1 M LiPF_6_ and EMC:EC:DMC at ratio 1:1:1	[61]
Fish scale	Fish scale mixed with KOH (1:1 ratio) followed by heating at 850 °C for 1 h under N_2_. Afterwards, washed with 1.0 M HCl	1980	Initial coulombic efficiency of 90%; RC of 500 and 480 mAh g^−1^ at a current density of 75 mA g^−1^ and discharge capacities of 224.7 and 232.5 mAh g^−1^1 at 2000 mA g^−1^1 after 75 cycles	N2224-TFSI *	[62]
Cherry pit	Conventional at 800 °C in for 2 h with KOH at 1:1 ratio	1171	98% for coulombic efficiency (upon 20 cycles); capacity retention of 94% (160 mAh g^−1^) upon 200 cycles; Energy density of about 450 Wh kg^−1^.	1 M LiPF_6_ and EC:DMC at ratio 1:1	[63]
Cherry pit	Conventional at 800 °C in for 2 h with H_3_PO_4_ at 1:1 ratio	1662	coulombic efficiency of 99% after 20 cycles; capacity retention higher than 96%. Energy density of about 450 Wh kg^−1^	1 M LiPF_6_ and EC:DMC at ratio 1:1	[63]
Banana peel	Pyrolysed at 1100 °C for 5 h and washed in 20% KOH at 70 °C for 2 h and 2 M HCl for 12 h	130.8	Initial coulombic efficiency of 55% at 50 mA g^−1^; RC of 1184 of 2nd at 50 mA g^-1^ and 790 of 11th cycle at 100 mA g^−1^; Rate capability of 243 mAh g^−1^ at 5 A g^−1^.	1 M LiPF6 in a 1:1:1 volume ratio of EC: DMC:DEC	[64]
Honey	700 °C for 2 h and then treated in 5% Hydrofluoric acid solution for 12 h	677.7	Initial coulombic efficiency of 61% at 100 mA g^−1^; RC of 1653 mAh g^−1^ of 1^st^ cycle and 1359 of 10th cycle at 100 mA g^−1^; Rate capability of 390 mAh g^−1^ at 5 A g^−1^.	LiPF_6_ (1M) in EC:DEC with ratio 1:1 (*v*/*v*)	[66]
Coffee	Conventional at 800 °C in for 2 h at N_2_	10	Electrode exhibited a remarkable anode performance with an RC of 285 mAh g^−1^ at 0.1 A g^−1^, an excellent capacity retention over 100 cycles and a coulombic efficiency nearly to 100%.	LiPF_6_ (1M ) in EC:DEC with ratio 1:1 (*v*/*v*)	[69]
Spongy pomelo peels	Carbonised at 900 °C in an argon-flowing for 3 h	114	Initial coulombic efficiency of 59.5% at 40 mA g^-1^; RC of 450 of 1st cycle at 40 mA g^−1^; Rate capability of 293 mAh g^-1^ at 0.32 A g^−1^.	LiPF_6_ (1M) in EC:DEC with ratio 1:1 (*v*/*v*)	[70]
Cotton cellulose	Carbon material mixed with elemental sulfur powder at ratio 1:1 and then carbonised at 600 °C under ar flow rate.	1265.9	Initial coulombic efficiency of 76% at 50 mA g^-1^; RC of 935 of 1st cycle at 50 mA g^−1^; Rate capability of 240 mAh g^−1^ at 2 A g^−1^.	1 M LiPF_6_ dissolved in EC:EMC (1:2:1 *v*/*v*)	[71]

* Ionic liquid in N-butyl,N,N,Ntriethylammonium bis(trifluoromethanesulfonyl) imide. EC—Ethylene carbonate. EMC—Ethyl methyl carbonate. DMC—Dimethyl carbonat.

**Table 3:** Electrochemical performance of some biomass CEs for double-layer supercapacitors.

Biomass Precursor	Pyrolysis Method and Optimal Condition	SSA (m^2^ g^−1^)	Main Electrochemical Findings	Electrolyte	Ref.
Cattle bone	Heated at 1100°C in for 1 h under N_2_ and washed with 1 M HCl	2096	The energy density of 109.9 W h kg^−1^ at a power density of 4.4 kW kg^−1^; energy density of 65.0 W h kg^−1^ at a power density of 81.5 kW kg^−1^; capacity retention of 96.4% after 5000 cycles.	EMIM-BF4	[9]
Puffed rice	Pre-carbonised at 500 °C for 1 h. Then, mixed with KOH and further activated at 850°C for 1 h under N_2_. Afterwards washed with 1 M HCl solution.	3326	Spe.Cap of 218 F g^−1^ at 80 A g^−1^; energy-density of 104 Wh kg^−1^ (53 Wh L^−1^)	6 M KOH	[52]
Reed membrane	Conventional - KOH		Spe.Cap of 353.6 F g^−1^ at 0.5 A g^−1^; Energy density of 57.7 Wh kg^−1^ at 10 kW kg^−1^; Rate capability of 184 F g−1 at 30 A g^−1^; 10000 cycles; capacitance retention of 91%	6 M KOH	[52]
Peanut shell	Impregnation with ZnCl_2_/biomass (4/1 ratio) for 12 h. Then, heated in Microwave oven at 600W for 20 min under N_2_.	1552	The energy density of 19.3 Wh kg^−1^ at a high power density of 1007 W kg^−1^. Spe.Cap reached 99 F g^−1^.	Et4NBF4/PC	[83]
Coconut shell	Biomass/ ZnCl_2_ at ratio 1:3 (w:w) in 50 mL of 3 M FeCl_3_ solution, then pyrolysied at 900 °C for 1 h under N_2_.	1874	Spe.Cap of 268 F g^−1^ at 1.0 A g^−1^; Energy density of 11.6 Wh kg^−1^ at 210 W kg^−1^; Rate capability of 76.9% at 10 A g^−1^; 5000 cycles; capacitance retention of 99.5%	6 M KOH	[84]
Coconut shell	Carbonised at 400 °C for 3 h under N_2_. Then, mixed with K_2_CO_3_ at ratio (1:2) and heated at 900 °C for 2 h and then, washed with HCl.	1506.2	Spe.Cap of 91.5 F g^−1^ at 0.2 A g^−1^; Energy density of 25.8 Wh kg^−1^ at 89 W kg^−1^; Rate capability of 72% at 50 A g^−1^; 20000 cycles; capacitance retention of 95%	1 M TEMABF4/propylene carbonate	[84]
Moringa oleifera stem	Biomass mixed with ZnCl_2_ (ratio 1:3) in 50 ml of 2 M FeCl_3_ solution for 2 h, then was heated at 800 °C for 2 h under N_2_. Then, washed with 2.0 M HCl.	2250	Spe.Cap of 283 F g^−1^ at 0.5 A g^−1^; Energy density of 11.6 Wh kg^−1^ at 95 W kg^−1^; 2000 cycles; capacitance retention of 82%	1.0 M Na_2_SO_4_ 1.0 M H_2_SO_4_	[85]
Bamboo	Biomass/KOH at 1:4 of ratio and pyrolysed at 750 °C under N_2_ and washed with HCl (6 wt%).	171.5	Spe.Cap of 318 F g^−1^ at 0.2 A g^−1^; Energy density of 42.1 Wh kg^−1^ at 210 W kg^−1^; Rate capability of 76.9% at 10 A g^−1^; 5000 cycles; capacitance retention of 99.5%	1 M H_2_SO_4_	[87]
Sodium alginate	Biomass mixed with CaSO_4_ and heated at 700 °C under Argon flow for 3 h. Sample soaked in 1 M HCl at 60 °C for 12 h.	1531.4	Spe.Cap of 424.6 F g^−1^ at the current density of 1 A g^−1^; capacitance retention of 90.4% and coulombic efficiency of 100%, respectively, after 20,000 charge-discharge cycles.	6 M KOH	[88]
Tea residues	Firstly, carbonised at 500 °C for 1 h in air. Then, biomass/KOH ratio of 1:4 by weight and heated at 700 °C under N_2_ and washed with 1 M HCl.	966.4	Spe.Cap of 162 F/g at 0.5 A/g; (cyclic capacitance retention of 121% over 5000 cycles); High cycle stability after sevral charge-discharge cycles.	1 M H2SO4	[89]
Tea leave	Biomass/KOH ratio of 1:2 by weight and heated at 900 °C for 1 h under Argon flow and washed with 1 M HCl. Afterwards, heated at 1200 °C for 60 min.	911.92	Spe.Cap of 167 F g^−1^ at 1.0 A g^−1^; Energy density of 47.86 Wh kg^−1^ at 1580.72 W kg^−1^; Rate capability of 81.42% at 30 A g^−1^; 16000 cycles; capacitance retention of 96.66%	6 M KOH 1 M Na_2_SO_4_	[90]
Cornhusk	5 g of biomass into 100 ml of 7% KOH solution at 80 °C for 4 h. Then, heated at 800 °C for 1 h under N_2_. Afterwards, washed with 1 M HCl solution.	928	Spe.Cap of 356 F g^−1^ at 1.0 A g^−1^; Energy density of 21 Wh kg^−1^ at 875 W kg^−1^; Rate capability of 88% at 10 A g^−1^; 2500 cycles; capacitance retention of 95%	6 M KOH	[91]
Cornhusk	5 g of biomass into 100 ml of 7% KOH solution at 80 °C for 4 h. Then, heated at 800 °C for 1 h under N_2_. Afterwards, washed with 1 M HCl solution.	928	Spe.Cap of 300 F g^−1^ at 20.0 A g^−1^; Energy density of 56 Wh kg^−1^ at 93 kW kg^−1^; Rate capability of 88% at 10 A g^−1^; 2500 cycles; capacitance retention of 95%	1 M Na2SO4	[91]
Shaddock skin	Biomass was mixed with ZnCl_2_ (ratio 1:2) in 50 ml 3 M FeCl_3_ solution at a solids loading of 5 wt.%. Then, heated at 900 °C for 2 h under Ar flow.	2327	Spe.Cap of 152 F g^−1^ at 1.0 A g^−1^; Energy density of 11 Wh kg^−1^ at 5600 W kg^−1^; Rate capability of 87% at 100 A g^−1^; 10000 cycles; capacitance retention of 97.6%	A mixture of EMI TFSI and EMI BF_4_	[92]
Bamboo shoot shells	4.0 g of biomass in 75 mL of 1 wt% H_2_SO_4_ solution were hydrothermally treated (HTC) at 200 °C for 24 h, and further heated at 800 °C with KOH (ratio of 1:2) for 1 h under N_2_ flow.	3300	Spe.Cap of 209 F g^−1^ at 0.5 A g^−1^; Coulombic efficiency of 100% at 10 mA g^−1^; Cycling stability performance of 95% after 10,000 cycles at 10 A/g.	6 M KOH	[93]
Alkali Lignin	Hydrothermally treated (HTC) at 180°C for 10 h, and further heated at 700 °C with KOH (ratio of 1:5) for 1 h under N_2_ flow	2486	Initial coulombic efficiency of 99.76% after 10,000 cycles; Spe.Cap of 384 F g^−1^ at 40 mA g^−1^; high energy density of 10.48 Wh kg^−1^.	6 M KOH	[94]
Macroalgae	HTC plus conventional with ZnCl_2_	~2000	coulombic efficiency ~ 100% 96% retention at 10 A/g after 10000 cycles; Spe.Cap of 202 F g^−1^ at 0.5 mA g^−1^; energy density of 7 Wh/kg and power density of 3000 W/kg	6 M KOH	[95]

ethylene carbonate (EC), dimethyl carbonate (DMC) and ethyl methyl carbonate (EMC).
